# MiR-139-5p negatively regulates PMP22 to repress cell proliferation by targeting the NF-κB signaling pathway in gastric cancer

**DOI:** 10.7150/ijbs.40338

**Published:** 2020-02-10

**Authors:** Jingjing Hou, Huiqin Zhuo, Xin Chen, Jia Cheng, Wei Zheng, Mengya Zhong, Jianchun Cai

**Affiliations:** 1 Department of Gastrointestinal Surgery, Zhongshan Hospital of Xiamen University, Xiamen, Fujian 361004, China; 2 Institute of Gastrointestinal Oncology, Medical college of Xiamen University, Xiamen, Fujian 361004, China; 3 Xiamen Municipal Key Laboratory of Gastrointestinal Oncology, Xiamen 361004, Fujian, China.

**Keywords:** PMP22, miR-139-5p, NF-κB, gastric cancer, cell proliferation

## Abstract

Gastric cancer (GC) is one of the most common malignant tumors worldwide. Peripheral myelin protein 22 (PMP22) is a 22-kDa tetraspan glycoprotein that is predominantly expressed by myelinating Schwann cells. However, recent studies have shown that PMP22 is closely related to cell proliferation and tumorigenesis in different cancers. In this study, we discovered a new miRNA that regulates PMP22 and gastric cancer cell prolifration. Our bioinformatics analysis suggested that there is a conserved miRNA recognition site for miR-139-5p on the 3' UTR of PMP22. Interestingly, our results showed overexpression of miR-139-5p significantly suppressed growth and prolifration in GC cells and inhibited tumor growth in nude mice xenografted with GC cells. MiR-139-5p suppressed the activity of a luciferase reporter containing the PMP22-3' UTR, and the ectopic expression of PMP22 rescued the miR-139-5p-mediated inhibition of cell proliferation in GC cells. Mechanistically, miR-139-5p may negatively regulate PMP22 to repress cell proliferation by targeting the NF-κB signaling pathway in gastric cancer. Finally, overexpression of miR-139-5p significantly inhibited tumor growth in nude mice xenografted with GC cells.and the miR-139-5p levels were inversely correlated with PMP22 expression in nude mice tumor. Taken together, our data suggest an important regulatory role of miR-139-5p in gastric cancer, suggesting that miR-139-5p and PMP22 might be important diagnostic or therapeutic targets for gastric cancer and other human diseases.

## Introduction

As one of the most common malignant tumors, gastric cancer (GC) is the fourth most commonly diagnosed cancer and the second leading cause of cancer deaths worldwide 1-3. Although there have been significant advancements in GC treatment, the outcome and survival of this cancer remain unclear. Additionally, the underlying molecular mechanisms in the process of GC are poorly understood. Thus, it is urgent to identify the cellular and molecular mechanisms of GC to improve clinical outcomes, develop early diagnostic markers, and facilitate improved therapeutic selection for GC patients 4, 5.

Peripheral myelin protein 22 (PMP22) is a 22-kDa tetraspan glycoprotein that is mainly expressed in myelinating Schwann cells and is closely related to Charcot-Marie-Tooth disease (CMT) 6, 7. Recently, more studies have described a role for PMP22 in cell proliferation and tumorigenesis in different cancers. However, the function of pmp22 in tumors remains controversial. Several studies indicate that PMP22 is a potential tumor suppressor 8-13, whereas some observations suggest a potential oncogenic function of PMP22 14-22. Few studies have examined the function and regulation of PMP22 in gastric cancer.

MicroRNAs (miRNAs) are important regulators and can function as oncogenes or tumor suppressors by targeting the 3' untranslated regions (UTR) of messenger RNA (mRNA) to induce mRNA degradation and suppress of translation 23, 24. Many stduies have showed that miRNAs play important roles in diverse biological processes and the dysfunction of these miRNAs contributes to the development of many types of cancer, including GC 25. Previous studies have shown that miR-9 interacts with the 3' untranslated region of PMP22 and downregulates PMP22 expression 26. Additionally, over-expression of miR-29a enhances the association of PMP22 RNA with Argonaute 2, a protein involved in miRNA function, and reduces the steady-state levels of PMP22 27, 28. However, few studies have examined the potential regulation of PMP22 by other RNA.

In this study, we found a new miRNA-miRNA29 that regulates PMP22 in GC cells. Our bioinformatics analysis identified a conserved miRNA recognition site for miR-139-5p on the 3' UTR of PMP22. Our results showed that miR-139-5p was down-regulated in GC, and the level of miR-139-5p was significantly lower in GC patients with late stage disease. The overexpression of miR-139-5p significantly suppressed growth and proliferation of GC cells and inhibited tumor growth in nude mice xenografted with GC cells. Expression of MiR-139-5p suppressed the activity of a luciferase reporter containing the pmp22-3' UTR, and the ectopic expression of PMP22 rescued the miR-139-5p-mediated inhibition of cell proliferation in GC cells. The miR-139-5p may negatively regulate PMP22 to repress cell proliferation by targeting the NF-KB signaling pathway. Consistently, qPCR assay showed that miR-139-5p was significantly reduced in clinical gastric cancer tissue and PMP22 mRNA and protein levels were up-regulated, suggesting that the down-regulation of miR-139-5p might be associated with the abnormal regulation of PMP22 and cell proliferation in gastric cancer development. Taken together, our data support an important regulatory role of miR-139-5p in gastric cancer, and suggest miR-139-5p and PMP22 might be important diagnostic or therapeutic targets for gastric cancers and other human diseases.

## Materials and Methods

### Cell culture and transent transfection

Human SGC7901, HGC27, BGC823, MKN45, MGC803 and 293T cells were obtained from Shanghai Academy of Sciences and the ATCC (Manassas, VA, USA), and the cells were maintained in DMEM medium (Gibco, Grand island, NY, USA) supplemented with 10% fetal bovine serum (Gibco, Grand island, NY, USA) and incubated in a humidified incubator at 37 ºC in 5% CO_2_. Plasmid DNA, miRNA mimics, and miRNA inhibitor (anti-sense oligonucleotides) transfections were performed with Lipofectamine 2000 reagent (Invitrogen, Carlsbad, CA, USA) according to the manufacturer's protocol.

### Colony formation assays

For colony formation assays, pLV-miR-139-5p or pLV-Ctrl were transfected into gastric cancer cells. The cells were seeded on six-well plates and maintained in DMEM containing 10% FBS for 2 weeks. Cells were fixed with methanol and stained with 0.5% crystal violet in 50% methanol for 1 hour and colonies larger than 100 μm in diameter were counted.

### Cell Proliferation Assays

The cell proliferation assay was performed using the Cell Counting Kit-8 (CCK-8; Dojindo, Japan) according to the manufacturer's instructions. The optical density (OD) value of each well was measured at 450 nm using a microplate spectrophotometer (Bio-Tek Instruments Inc., Winooski, VT, USA). For the colony formation assay, the methods were as previously described 20.

### Dual-Luciferase Reporter Assay

PMP22 was identified as a miR-139-5p target in TargetScan7.1 (http://www.targetscan.org/vert_71/). The wild-type (Wt) and mutant (Mut) DNA sequences of PMP22 were custom synthesized by GeneChem Co., Ltd (Shanghai, P.R. China). We synthesized the 901-1100 sequence of PMP22, as well as a variant sequence (Mut) in which bases were replaced at positions 1011-1018 (changed from UGACAUC to CATGCAT). These two sequences were separately cloned into the pGL3-control vector (Ambion, Austin, TX, USA). Cells were cotransfected with 100 ng of the above-described luciferase-PMP22 mRNA 3'-UTR constructs and with 50 nM of either miR-139-5p mimics or miR-NC together with the Renilla luciferase construct, respectively, using Lipofectamine 3000 (Invitrogen). After 48 hours, the cells were collected, and the luciferase and Renilla luciferase activities were determined using the Dual-Luciferase Reporter Assay System (Promega, Madison, WI, USA). Firefly luciferase activity was normalized to Renilla luciferase activity.

### Animal experiments

All experimental procedures involving animals were performed in accordance with animal protocols approved by the Institutional Animal Use and Care Committee of Xiamen University. For xenograft tumor growth, LV-miR-139-5P or LV-Ctrl-infected SGC7901 cells (1 × 10^6^) were suspended in 100 µL PBS and then injected into the right side of the posterior flank of BALB/c athymic nude mouse at 5 to 6 wk. Beginning from the 7th day after the injection, the size of the tumor was measured every 5 days by a Vernier caliper along two perpendicular axes. The volume of the tumors was calculated with the formula: volume (mm^3^) = length (mm) × width (mm)^2^ ×0.52. Thirty days after the injection, the mice were sacrificed and the tumors were dissected for further analyses.

### Clinical samples

All clinical samples were collected with the informed consent of the patients and study protocols that were in accordance with the ethical guidelines of the Declaration of Helsinki (1975) and were approved by the Institutional Medical Ethics Committee of Xiamen University. GC pathological diagnosis was verified by at least two pathologists. A total of 48 sets of human GC specimens and paired adjacent epithelial tissues were obtained from the Zhongshan Hospital of Xiamen University from 25 July 2016 to June 2019.

### Plasmid construction

To construct lentiviral vectors overexpressing miR-139-5p, the human miR-139-5p gene DNA fragment was PCR amplified from human SGC7901 cell genomic DNA The PCR-amplified fragment were inserted into a lentiviral vector pLV-EF1α-MCS-IRES-Puro (pLV-ctrl) to generate pLV-miR-139-5p. Viral vector pLV-139-5p or pLV-ctrl as well as three lentivirus packaging plasmids (pMDL, pVSVG, and pREV) were co-transfected into HEK293T cells. Media containing lentiviruses (pLV-miR-139-5p and pLV-ctrl) were collected every 24 h for three times and the lentiviruses were purified by ultra-speed centrifugation. Full-length cDNA encoding human PMP22 was amplified by PCR, and the PCR product was sub-cloned into pBOBI and pCMV-HA vectors to obtain PMP22 overexpressing plasmids. Luciferase reporter plasmids PGL-3-NF-κB, pLV-EF1α-MCS-IRES-Puro, pMDL, pVSVG and pREV were kindly provided by Professor Jiahuai Han (Xiamen University, Xiamen, China). All constructs derived from PCR products were verified by DNA sequencing.

### RNA interference

The pLKO.1 lentiviral vector was used to express short hairpin RNA directed against the PMP22 or LacZ control sequence (GTCTCCGAACGTGTCACGTT). Oligonucleotides targeting PMP22 (PMP22 shRNA-1, 5'- CCAAACTCAAACCAAACCAAA -3'; PMP22 shRNA-2, 5'- CGGTGTCATCTATGTGATCTT -3') were cloned into the pLKO.1 lentiviral vector. Recombinant lentiviral plasmids were cotransfected into 293T cells with the packaging plasmids VSV-G, RSV-REV, and pMDL. After 48 h the viral supernatants were passed through 0.45-μm filters and used to infect target cells in the presence of 8 μg/ml polybrene (Sigma-Aldrich).

### Real-time quantitative PCR (qPCR) analyses of mRNAs and miRNAs

For qPCR analyses of mRNA, reverse transcription was performed with TRIzol (Invitrogen)-extracted total RNAs using a ReverTra Ace-α^®^ Kit as instructed (Cat.# FSQ-101, Toyobo, Tokyo, Japan). RT-PCR was performed using the SYBR Green Real-Time PCR Master Mix (Cat.# QPK-212, Toyobo) and the Step One Plus Real-Time PCR system (Applied Biosystems Inc., Foster City, CA, USA) using appropriate primer pairs as listed in Table 1, according to the manufacturers' protocols and with GAPDH rRNA as a control.

For miRNAs, qPCR was performed with the stem-loop primers as reported previously 29, 30. U6 RNA served as an internal control. The miRNA-specific stem-loop primers are listed in Table 1. RT-PCR was performed with total RNA samples, using universal primer and miRNA-specific reverse LNA-primers as listed in Table 1, with U6 RNA as an internal control.

### Statistical analysis

All statistical tests were performed using Graphpad Prism 6.0 (GraphPad Software Inc, San Diego, CA, USA). Values represent the mean ± SD for at least three independent experiments. One-way ANOVA with Bonferroni's post-test was used for multiple comparisons and the Student's t test (two-tailed) was used for pair-wise comparisons. Correlation analyses were performed with Pearson's test. P values < 0.05 were considered statistically significant.

## Results

### 3.1. miRNA139-5p predicted to regulate PMP22

**Figure 1:**
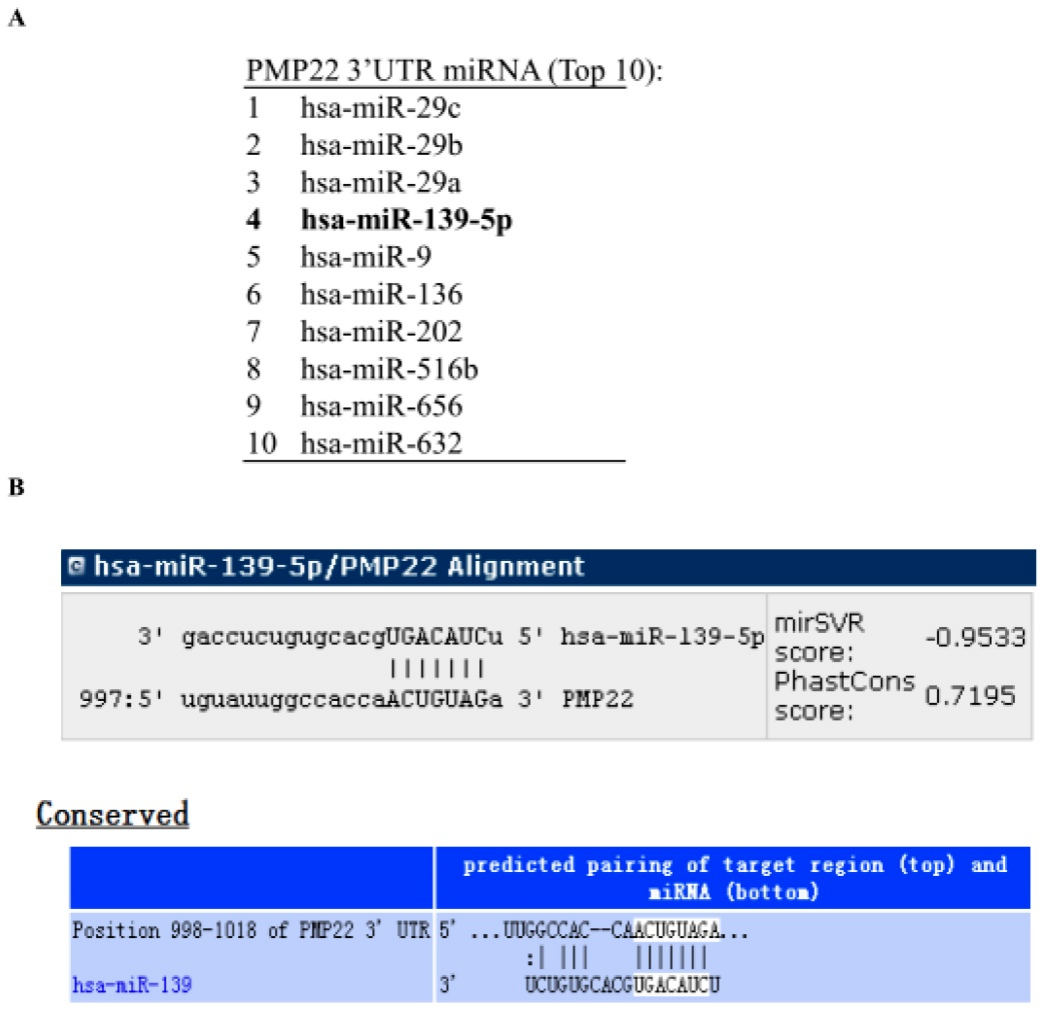
** miRNA139-5p might regulate PMP22 according to prediction software.** (A) The top 10 miRNAs predicted to bind to PMP22 based on analysis with different prediction software packages (additional information in Table 2). (B) The predicted binding site of miR-139-5p in the 3' UTR of PMP22.

To identify miRNAs that might target PMP22, we analyzed the 3' UTR sequence of human PMP22 with prediction software including miRanda (http://www.microrna.org), miRDB (http://www.mirdb.org), and TargetScan (http://www.targetscan.org). The miRNAs predicted to bind to PMP22 are listed in Table 2, and the top 10 miRNAs are shown in Fig. 1A. Our bioinformatics analysis identified a putative *microRNA* response element (MRE) on human PMP22 3'UTR (Fig. 1B), suggesting that the miRNA139-5p might regulate PMP22.

### 3.2 miR-139-5p inhibits GC cell proliferation and growth in vitro

**Figure 2:**
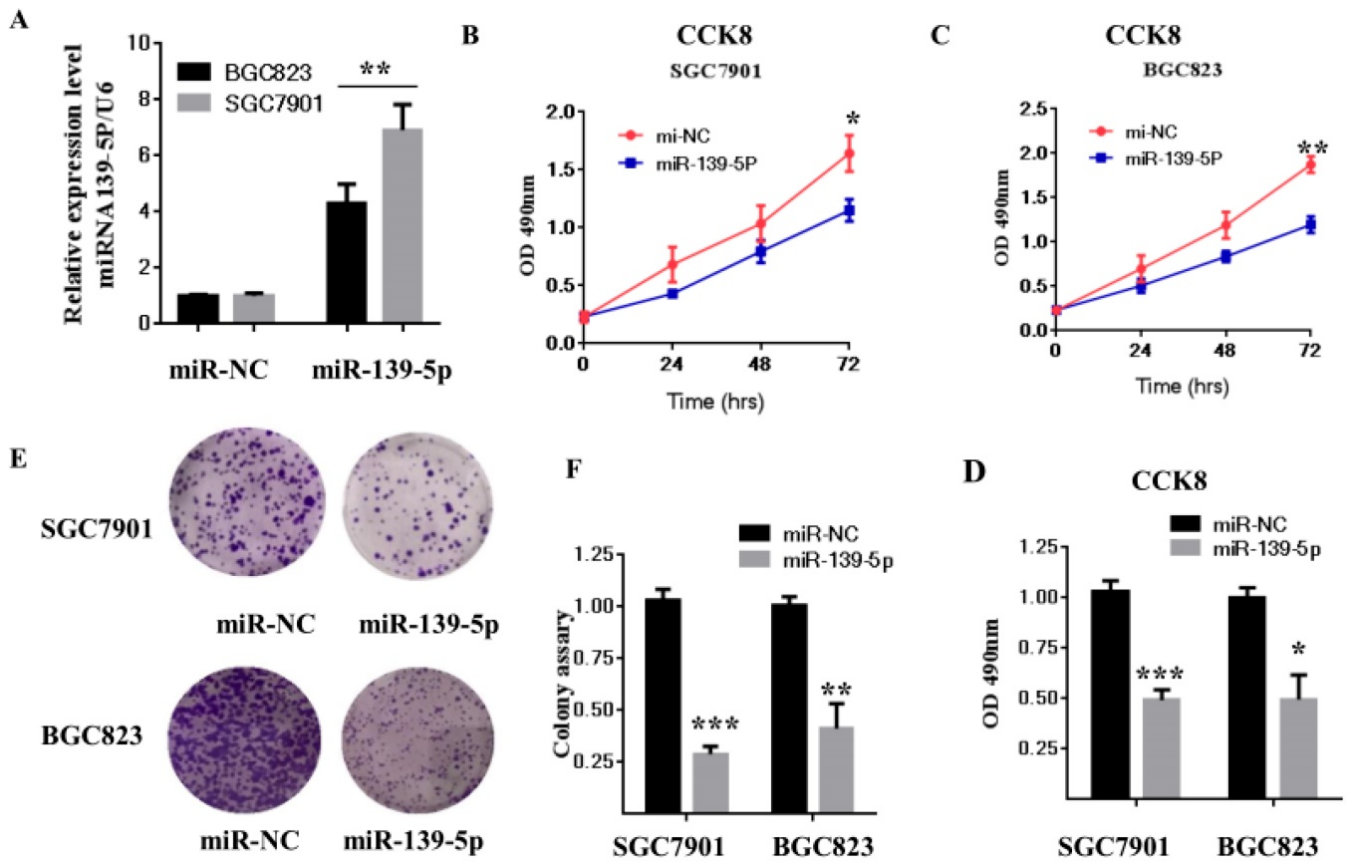
** The miR-139-5p inhibits GC cell proliferation in vitro.** (A) BGC823 and MGC803 cells were transfected with miR-139-5p mimic or its negative control (miR-NC), and the expression level of miR-139-5p was measured by qRT-PCR. (B-C) Cell viability was determined in BGC823 and MGC803 cells transfected with miR-139-5p mimic or miR-NC by CCK8 assay. (D) Statistical analysis of the CCK8 assay results after 72h. (E) Cell proliferation was detected by colony formation assay. (F) Statistical analysis of data presented in E. Results are representative of three independent experiments, and the error bars represent the SD. *p < 0.05, ** p < 0.01, *** p < 0.001.

Previous studies reported that miR-139-5p is suppressed in several types of tumors, including glioblastoma multiforme, human nasopharyngeal carcinoma, chronic myeloid leukemia (CML), and ovarian cancer 31-34. To evaluate miR-139-5p's biological function in GC, SGC7901 and BGC823 cells were transfected with miR-139-5p mimics or a negative control (miR-NC), and then cell proliferation and colony formation were measured. As shown in Fig. 2A, qRT-PCR results showed that SGC7901 and BGC823 cells transfected with miR-139-5p mimics exhibited significantly enhanced miR-139-5p expression compared with the levels in cells transfected with miR-NC.

We next used the CCK-8 assay to assess the effects of miR-139-5p on cell proliferation. The result showed that miR-139-5p overexpression significantly decreased proliferation of SGC7901 and BGC823 cells (Fig. 2B-D). Consistent with this result, the colony formation assay confirmed that miR-139-5p overexpression significantly decreased cell proliferation (Fig. 2E-F). Taken together, these data clearly support inhibition by miR-139-5p of GC cell proliferation and growth.

### 3.3 PMP22 is a direct target of miR-139-5p

**Figure 3:**
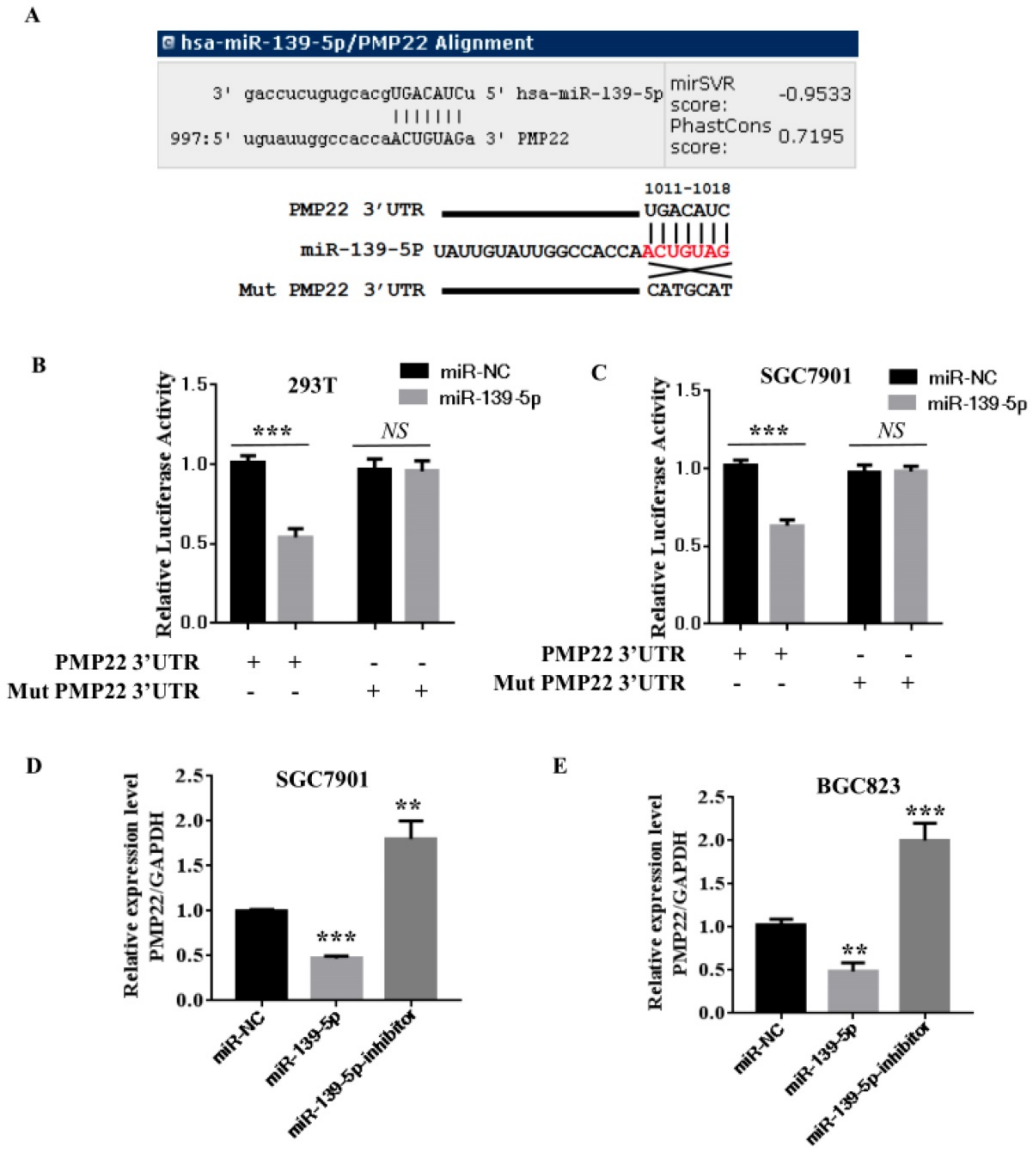
** PMP22 is a direct target of miR-139-5p in GC cells.** (A) The putative miR-139-5p binding sites and mutant (Mut) 3'-untranslated region (3'-UTR) PMP22 sequences are shown. The replaced site is underlined. WT, wild type; MT, mutant type. (B-C) Relative luciferase activity was measured in 293T (B) or SGC7901(C) cells cotransfected with WT/MT-PMP22-3'UTR reporter plasmid and miR-139-5p mimic or miR-NC. (D-E) PMP22 mRNA and protein expression levels were measured in SGC7901 or BGC823 cells transfected with miR-NC, miR-139-5p mimic, and miR-139-5p-inhibitor. Glyceraldehyde 3-phosphate dehydrogenase (GAPDH) was used as an internal control, **p < 0.01.

Our bioinformatics analysis identified a putative miRNA MRE in the 3'UTR of human PMP22 (Fig. 3**A**). To investigate the potential targeting of PMP22 by miR-139-5p, a luciferase activity assay was designed. We constructed a PMP22-3' UTR-luciferase reporter plasmid and a reporter plasmid with a mutated 3' UTR sequence (Mut) (Fig. 3A). We first used qPCR analyses to confirm that miR-139-5p-transfected cells showed significantly increased miR-139-5p expression (p < 0.001 and p < 0.01, Fig. 3B-C, left panel). Expression of miR-139-5p in 293T and SGC7901 cells obviously inhibited the luciferase activity of the PMP22-3UTR reporter, but not that of the Mut reporter with the altered sequence (Fig. 3B-C). Additionally, our qPCR analyses indicated that, compared to the control transfected group, transfection of 293T and SGC7901 cells with miR-139-5p significantly increased miR- miR-139-5p expression (p < 0.001 and p < 0.01, Fig. 3B-C, left panel). Next, we examined the mRNA expression of PMP22 in SGC7901 and BGC823 cells transfected with the indicated miRNA. As shown in Fig. 3C, miR-139-5p mimics significantly decreased PMP22 mRNA expression, and the miR-139-5p inhibitor significantly increased PMP22 mRNA expression in GC cells (Fig. 3D-E). Together, these data suggest that PMP22 mRNA was subjected to post-transcriptional control of miR-139-5p by targeting the PMP22-3' UTR.

### 3.4 Overexpression of PMP22 ablates the inhibitory effects of miR-139-5p in gastric cancer cells

**Figure 4:**
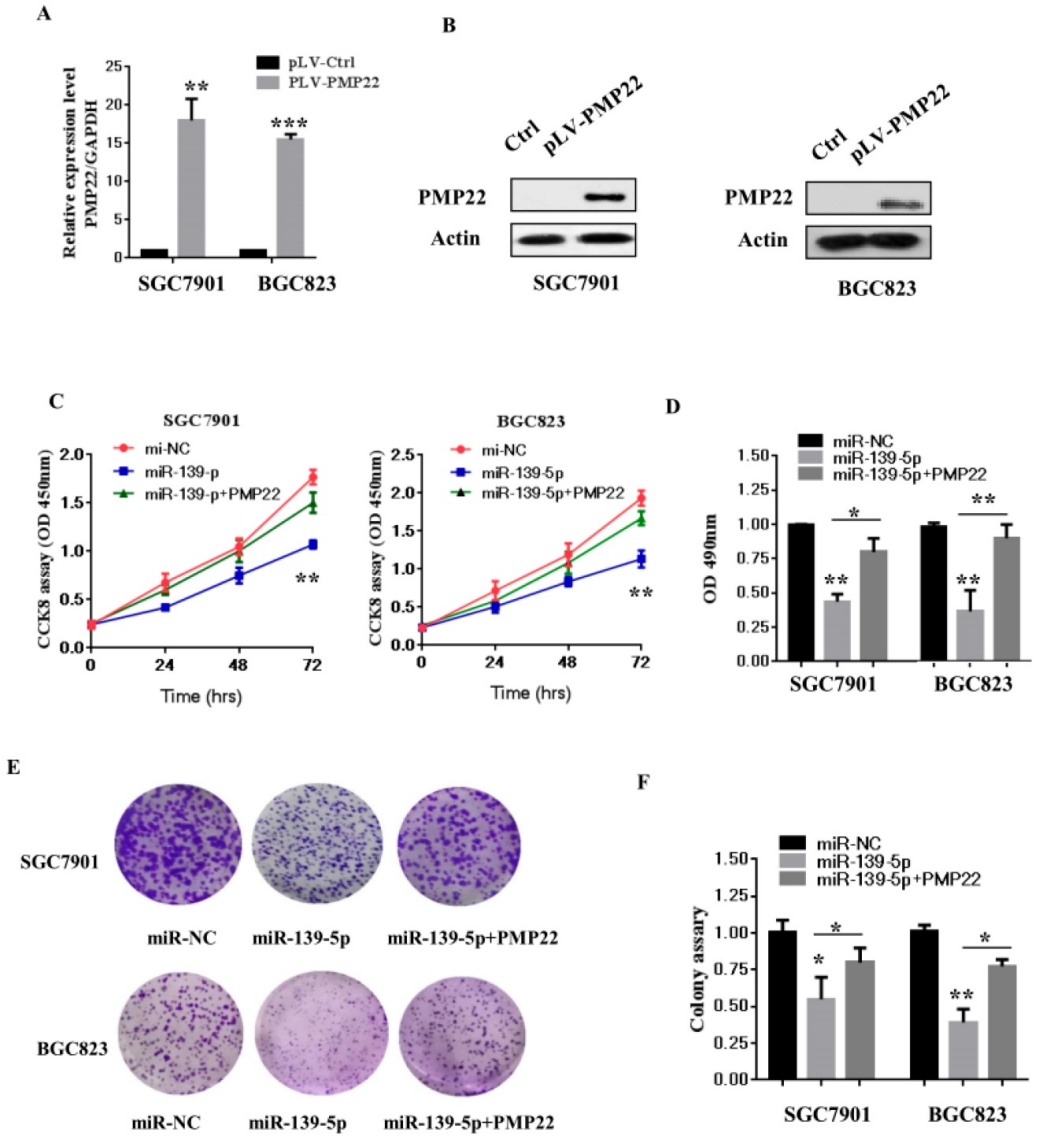
** Overexpression of PMP22 ablates the inhibitory effects of miR-139-5p in GC cells.** (A) The q-RT-PCR results show the expression of PMP22 in the SGC7901 and BGC823 cells infected with PLV-PMP22 plasmids. (B) SGC7901 and BGC823 cells were infected with PLV-PMP22 plasmids, and the cells were harvested for western blot analysis with antibodies against PMP22 and Actin. (C) Cell proliferation was determined by CCK-8 assay. The ectopic expression of PMP22 rescued the miR-139-5p-mediated inhibition of cell proliferation in SGC7901 and BGC cells. (D) Statistical analysis of the CCK8 assay results at 72 h in C. (E) The ectopic expression of PMP22 rescued the miR-139-5p-mediated inhibition of cell proliferation in SGC7901 and BGC cells. (F) Statistical analysis of E. *p < 0.05, and **p < 0.01, ***p < 0.001.

To examine whether miR-139-5p affects GC cell growth and proliferation via targeting PMP22, we next constructed a virus packaging plasmid, PLV-PMP22. SGC7901 and BGC823 cells were infected with lentiviruses expressing pLV-PMP22, and the q-PCR results confirmed significantly increased expression of PMP22 mRNA after infection with the PMP22-expressing virus (Fig. 4A). The western blot results showed that the protein level of PMP22 also significantly increased after infection with the PMP22-expressing virus (Fig. 4B). We next asked if PMP22 overexpression could ablate the inhibitory effects of miR-139-5p in SGC7901 and BGC823 cells. As expected, the CCK 8 assay showed that the ectopic expression of PMP22 rescued the miR-139-5p-mediated inhibition of cell proliferation in SGC7901 and BGC823 cells (Fig. 4C). In addition, PMP2 overexpression reversed the inhibitory effects of miR-139-5p on colony formation (Fig. 4E-F). These results suggested that miR-139-5p exerts a suppressive role in GC by repressing PMP22.

### 3.5 MiR-139-5p represses PMP22 by inhibiting the NF-κB pathway

**Figure 5:**
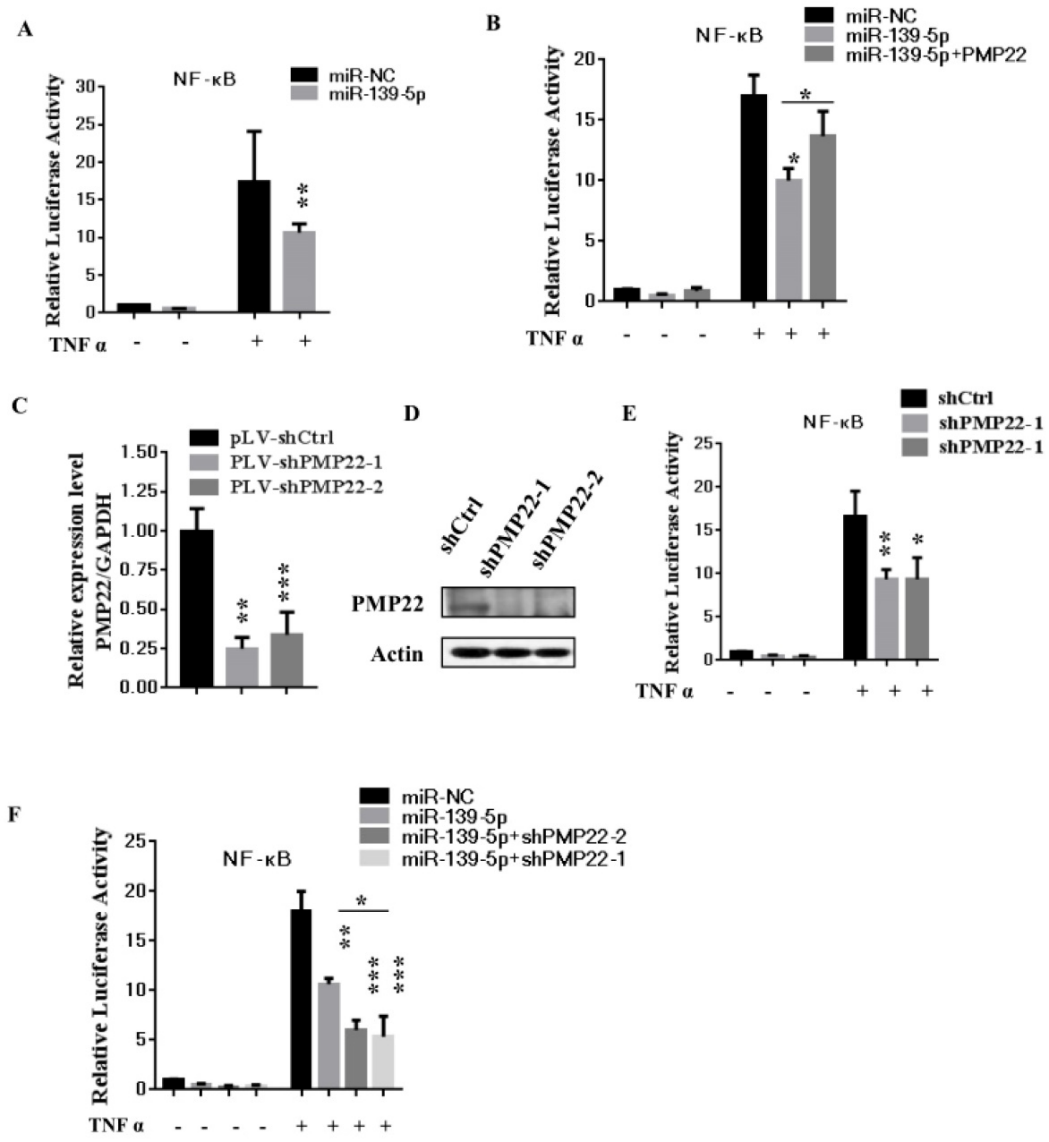
** MiR-139-5p represses PMP22 by inhibting the NF-κB pathway**. (A) Luciferase reporter assays of NF-κB. SGC7901 cells were transfected with miR-139-5p. After 48h, cells were treated with or without TNFα, and then luciferase reporter assays were performed. (B) Luciferase reporter assays of NF-κB. Ectopic expression of PMP22 rescued the miR-139-5p-mediated inhibition of NF-κB reporter gene activity in SGC7901 cells. (C) The q-RT-PCR analysis of the expression of PMP22 in SGC7901 cells infected with PLV-shPMP22 plasmids. (D) SGC7901 cells were infected with PLV-shPMP22 plasmids, and the cells were harvested for western blot analysis with antibodies against PMP22 and Actin. (E) NF-κB reporter gene activity was detected in the SGC7901-shPMP22 cell line after TNFα stimulation. (F) Luciferase reporter assays. PMP22 knockdown increased the inhibitory effects induced by miR-139-5p on NF-κB reporter gene activity in SGC7901 cells.

The NF-κB pathway promotes cell proliferation and oncogenesis by protecting cells from apoptosis 35, 36. We speculated that miR-139-5p might regulate PMP22 through the NF-κB pathway. Therefore, we examined the effects of miR-139-5p and PMP22 on the transcriptional activities of NF-κB using a NF-κB-luc reporter gene assay with in the presence or absence of TNF-α, whose signals are primarily mediated through NF-κB 35. As shown in Fig. 5A, NF-κB-mediated gene activation was inhibited by miR-139-5p in cells with or without TNF-α, with a 1.8-fold decrease in NF-κB reporter gene activation compared to that of the control. SGC7901 cells were then infected with lentiviruses expressing either the control construct or pLV-shPMP22 and NF-κB reporter gene activity was examined. As shown in Fig. 5B and 5C, the q-PCR results and western blot results confirmed a significant decrease in PMP22 mRNA and protein expression after infection with lentiviruses expressing pLV-shPMP22. NF-κB reporter gene activity was also inhibited after PMP22 knockdown with TNFα stimulation (Fig. 5D). We next asked if the overexpression of PMP22 could ablate the inhibitory effects of miR-139-5p on NF-κB reporter gene activity. The results are shown in Fig. 5E, and revealed that PMP22 ablates the inhibitory effects induced by miR-139-5p on NF-κB reporter gene activity, while PMP22 knockdown increased the inhibitory effects induced by miR-139-5p on NF-κB reporter gene activity (Fig. 5F). Together these results indicate that overexpression of miR-139-5p may suppress PMP22 via inhibition of the NF-κB pathway.

### 3.6 MiR -139-5p suppresses tumorigenicity in vivo in nude mice

**Figure 6:**
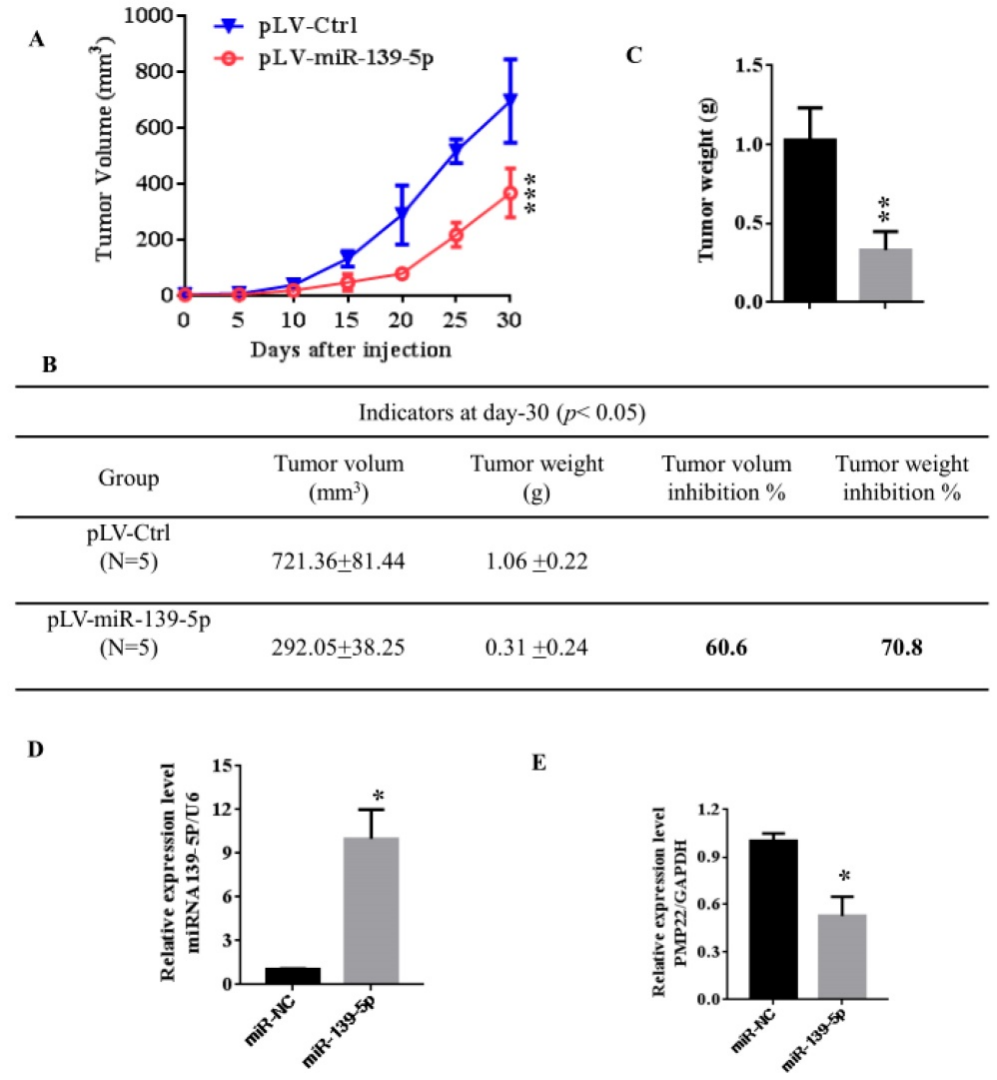
** MiR-139-5p suppresses tumorigenicity in vivo in nude mice.** (A) The tumor growth curve. Lentivirus-infected SGC7901 cells (LV-miR-139-5p or LV-Ctrl-infected SGC7901 cells) were injected into the right posterior flank of nude BALB/c mice. Thirty days after implantation, we observed tumor growth. (B) Photographs of the dissected xenograft tumors from various groups of nude mice treated as indicated. (C) Statistical analysis of the tumor weight of each group. *p < 0.05, **p < 0.01. (D) q-PCR assay. The miR-139-5p and PMP22 mRNA expression levels in SGC7901 xenograft tumors (n=5). *p < 0.05, and **p < 0.01, ***p < 0.001.

**Figure 7:**
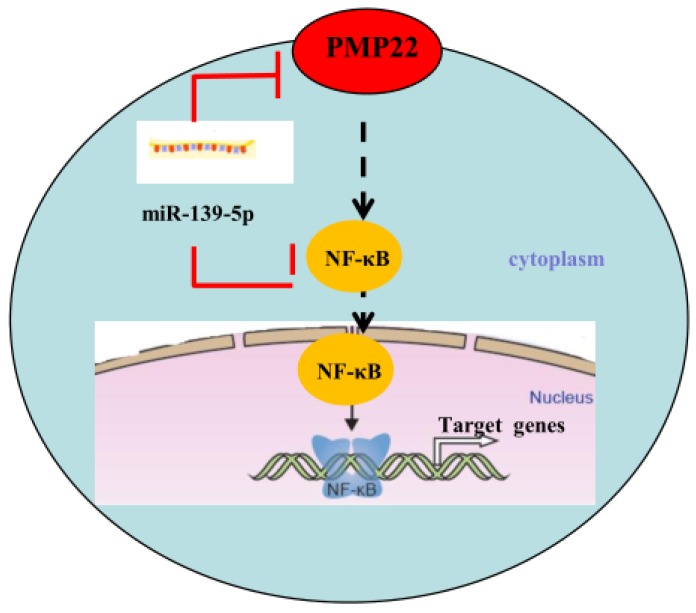
** Proposed working model of miR-139-5p and PMP22 in the regulation of gastric cancer.** MiR-139-5p negatively regulates PMP22 to repress cell proliferation by targeting the NF-κB signaling pathway in gastric cancer.

To further confirm the above findings, an in vivo model was used. Lentivirus-infected SGC7901 cells (LV- miR-139-5p or LV-Ctrl-infected SGC7901 cells) were injected into the right posterior flank of nude BALB/c mice. Thirty days after implantation, we observed that the sizes of SGC7901-mi-RNA-139-5p cell-derived xenograft tumors were significantly smaller than those of SGC7901-Ctrl cell-derived tumors (Fig. 6A-C). The tumor weight of the miRNA-139-5p group was 30% lower than the control group (p < 0.01), the volume inhibition rate was 60.6%, and the weight inhibition was 70.8% compared with the control group (Fig. 6B). These results demonstrated that miRNA-139-5p inhibits the tumorigenicity of gastric cancer cells in a nude mouse xenograft model. We also performed qPCR to detect PMP22 expression in randomly selected xenograft mouse tumors and found that miR-139-5p-overexpressing tumors expressed lower levels of PMP22 (Fig. 7D).

## Discussion

Gastric carcinoma (GC) is one of the most common malignant tumors. Although many studies have reported the regulation and treatment of gastric cancer, the underlying molecular mechanisms of GC remain unclear 1-3. Here, we describe a new miRNA that regulates PMP22 and gastric cancer cell proliferation. Our results showed that: (1) The expression of miR-139-5p is downregulated in gastric cancer tissues and cancer lines (Fig. 1); (2) miR-139-5p inhibits GC cell proliferation in vitro (Fig. 2); (3) PMP22 is a direct target of miR-139-5p in GC cells (Fig. 3); (4) Overexpression of PMP22 ablates the inhibitory effects of miR-139-5p in GC cells (Fig. 4); (5) MiR-139-5p represses PMP22 by inhibiting the NF-κB pathway (Fig. 5), and (6) miR-139-5p suppresses tumorigenicity in vivo in nude mice (Fig. 6). We constructed a possible pattern diagram and present the molecular mechanism by which miR-139-5p may regulate cell proliferation via PMP22 (Fig. 7). Collectively, these results demonstrate an important regulatory role of miR-139-5p in gastric cancer. The miR-139-5p might suppress the expression of PMP22 and tumor proliferation by inhibition of NF-κB signaling in gastric cancer, suggesting miR-139-5p and PMP22 might be important diagnostic or therapeutic targets for gastric cancer and other human diseases.

PMP22, also known as gas-3, is a tetraspan glycoprotein with proposed roles in peripheral nerve myelin formation, cell-cell interactions, and cell proliferation 6, 7. Most research on PMP22 proteins has focused on the nervous system. However, recent several studies show that PMP22 regulates tumor development, metastasis, and invasion in different cancers 8-19, 21. At present, there is no clear evidence of PMP22 acting as an oncogene or as a tumor suppressor gene. PMP22 is directly associated with progression and metastasis of breast cancer and the expression of PMP22 is decreased in breast cancer 12, 13. Down-regulated PMP22 expression has been reported for urethan-induced lung tumors in mice and two malignant histiocytoma cell lines 11. In contrast, Maaike and coworkers observed frequent amplification and overexpression of PMP22 in high-grade osteosarcoma and high-grade glioma 20, 21. Our previous studies showed a requirement for PMP22 in self-renewal and chemoresistance in gastric cancer 22, however, there are few studies on the regulation of PMP22 in gastric cancer.

MicroRNAs (miRNAs) are important regulators and can function as oncogenes or tumor suppressors 23, 24. Previous studies have shown that miR-9 and miR-29a interact with the 3' untranslated region of PMP22 and downregulate PMP22 expression in the nervous system and nerve cells 26-28. However, the regulation of PMP22 by microRNA in GC is not clear, and there is an urgent need to clarify the mechanism by which PMP22 is modulated by these newly identified miRNAs. In our study, we predicted the binding of PMP22 by miRNA and verified miR-139-5p as a novel regulator of PMP22 in GC. MiR-139-5p is highly important, with extensive function in human tumorigenesis and development 31. This miRNA has been related to several tumor types, including human nasopharyngeal carcinoma, chronic myeloid leukemia (CML), ovarian cancer, gastric cancer, breast cancer, GBM, and glioblastoma 31-34, 37-39. The MicroRNA-139-5p inhibits cell proliferation and invasion by targeting RHO-associated coiled-Coil-Containing Protein Kinase 2 in Ovarian Cancer 33, which acts as a tumor suppressor by targeting ELTD1 and regulating the cell cycle in glioblastoma multiforme 31, affects cisplatin sensitivity in human nasopharyngeal carcinoma cells by regulating the epithelial-to-mesenchymal transition 32, regulates proliferation of hematopoietic progenitors, and is repressed during BCR-ABL-mediated leukemogenesis 32, and controls translation in myeloid leukemia through EIF4G2 40. Consistent with previous study, our observations show that miR-139-5p is obviously deregulated, and is expressed inversely with PMP22 in GC patients and cell lines. MiR-139-5p suppresses the proliferation of GC cells by inhibiting PMP22 expression and the NF-κB signaling pathway.

The NF-κB signaling pathway is closely related to cell proliferation and oncogenesis. In response to numerous stimuli including tumor necrosis factor alpha (TNF-α) and bacterial lipopolysaccharide (LPS), NF-κB is activated and translocated into the nucleus from the cytoplasm, where it acts as a transcription factor to regulate its downstream target genes 35, 36, 41-43. In our study, miR-139-5p and PMP22 knockdown both suppress the NF-κB signaling pathway, and PMP22 overexpression rescued the miR-139-5p-induced NF-κB reporter activity, suggesting that PMP22 may enhance tumor proliferation through the NF-κB signaling pathway. Interesting, PMP22 is a predicted membrane protein, so how PMP22 regulates the NF-κB signaling pathway requires further study.

Taken together, our data suggest an important regulatory role of miR-139-5p in gastric cancer, and indicate that miR-139-5p and PMP22 might be important diagnostic or therapeutic targets for gastric cancers and other human diseases.

## Figures and Tables

**Table 1:** The sequences of primers used in qRT-PCR

Primer	Sequence (5'-3')
miR-139-5p (F)	GCCTCTACAGTGCACGTGTCTC
miR-139-5p (R)	CGCTGTTCTCATCTGTCTCGC
U6 (F)	CTCGCTTCGGCAGCACA
U6 (R)	AACGCTTCACGAATTTGCGT
PMP22 (F)	CTGGTCTGTGCGTGATGAGTG
PMP22 (R)	ATGTAGGCGAAACCGTAGGAG
GAPDH (F)	TCTCCTCTGACTTCAACAGCGA
GAPDH (R)	GTCCACCACCCTGTTGCTGT

**Table 2:** miRNAs predicted to bind to 3'-UTR of Human PMP22 by different predictive software (top 28).

	miRNA	StemLoopID	DIANAmT	miRanda	miRDB	miRWalk	RNAhybrid	PICTAR4	PICTAR5	PITA	RNA22	Targetscan	SUM
PMP22	hsa-miR-29c	hsa-mir-29c	1	0	1	1	1	1	1	1	0	1	8
PMP22	hsa-miR-29b	hsa-mir-29b-2	1	0	1	1	1	1	1	1	0	1	8
PMP22	hsa-miR-29a	hsa-mir-29a	1	0	1	1	1	1	1	0	0	1	7
**PMP22**	**hsa-miR-139-5p**	**hsa-mir-139**	**1**	**0**	**1**	**1**	**1**	**0**	**1**	**0**	**0**	**1**	**6**
PMP22	hsa-miR-9	hsa-mir-9-3	1	0	1	1	1	0	1	0	0	1	6
PMP22	hsa-miR-136	hsa-mir-136	1	0	1	1	0	0	1	0	0	1	5
PMP22	hsa-miR-202	hsa-mir-202	1	0	0	1	0	0	1	1	0	1	5
PMP22	hsa-miR-516b	hsa-mir-516b-1	1	0	1	1	0	0	1	0	0	1	5
PMP22	hsa-miR-656	hsa-mir-656	1	0	1	1	0	0	1	0	0	1	5
PMP22	hsa-miR-632	hsa-mir-632	1	0	1	1	0	0	1	0	0	1	5
PMP22	hsa-miR-432	hsa-mir-432	1	0	0	1	0	0	1	1	0	1	5
PMP22	hsa-miR-495	hsa-mir-495	1	0	1	1	0	0	1	0	0	1	5
PMP22	hsa-miR-505	hsa-mir-505	1	0	1	1	0	0	1	0	0	1	5
PMP22	hsa-miR-767-5p	hsa-mir-767	1	0	1	1	0	0	1	0	0	1	5
PMP22	hsa-miR-485-5p	hsa-mir-485	1	0	1	1	0	0	1	0	0	1	5
PMP22	hsa-miR-220b	hsa-mir-220b	0	1	0	1	0	0	1	0	0	1	4
PMP22	hsa-miR-582-5p	hsa-mir-582	1	1	0	1	0	0	0	0	0	1	4
PMP22	hsa-miR-377	hsa-mir-377	1	1	0	1	0	0	0	0	0	1	4
PMP22	hsa-miR-129-5p	hsa-mir-129-2	1	0	0	1	0	0	1	0	0	1	4
PMP22	hsa-miR-300	hsa-mir-300	1	1	0	1	0	0	0	0	0	1	4
PMP22	hsa-miR-662	hsa-mir-662	0	1	0	1	0	0	1	0	0	1	4
PMP22	hsa-miR-576-3p	hsa-mir-576	1	0	0	1	0	0	1	0	0	1	4
PMP22	hsa-miR-488	hsa-mir-488	1	0	0	1	0	0	1	0	0	1	4
PMP22	hsa-miR-1324	hsa-mir-1324	0	0	1	1	0	0	1	0	0	1	4
PMP22	hsa-miR-508-5p	hsa-mir-508	1	0	0	1	0	0	1	0	0	1	4
PMP22	hsa-miR-15b	hsa-mir-15b	1	1	0	1	0	0	0	0	0	1	4
PMP22	hsa-miR-924	hsa-mir-924	1	1	0	1	0	0	0	0	0	1	4
PMP22	hsa-miR-518a-5p	hsa-mir-518a-2	1	1	0	1	0	0	0	0	0	1	4

## References

[1] Parkin DM, Bray F, Ferlay J, Pisani P (2005). Global cancer statistics, 2002. CA Cancer J Clin.

[2] Danaei G, Vander Hoorn S, Lopez AD, Murray CJ, Ezzati M (2005). Causes of cancer in the world: comparative risk assessment of nine behavioural and environmental risk factors. Lancet.

[3] Ferlay J, Soerjomataram I, Dikshit R, Eser S, Mathers C, Rebelo M (2012). Cancer incidence and mortality worldwide: sources, methods and major patterns in GLOBOCAN 2012. Int J Cancer.

[4] Nagini S (2012). Carcinoma of the stomach: A review of epidemiology, pathogenesis, molecular genetics and chemoprevention. World J Gastrointest Oncol.

[5] Jiang G, Wen L, Zheng H, Jian Z, Deng W (2016). miR-204-5p targeting SIRT1 regulates hepatocellular carcinoma progression. Cell Biochem Funct.

[6] Patel PI, Roa BB, Welcher AA, Schoener-Scott R, Trask BJ, Pentao L (1992). The gene for the peripheral myelin protein PMP-22 is a candidate for Charcot-Marie-Tooth disease type 1A. Nat Genet.

[7] Snipes GJ, Suter U, Welcher AA, Shooter EM (1992). Characterization of a novel peripheral nervous system myelin protein (PMP-22/SR13). J Cell Biol.

[8] Wang L, Mear JP, Kuan CY, Colbert MC (2005). Retinoic acid induces CDK inhibitors and growth arrest specific (Gas) genes in neural crest cells. Dev Growth Differ.

[9] Winslow S, Leandersson K, Larsson C (2013). Regulation of PMP22 mRNA by G3BP1 affects cell proliferation in breast cancer cells. Mol Cancer.

[10] Karlsson C, Afrakhte M, Westermark B, Paulsson Y (1999). Elevated level of gas3 gene expression is correlated with G0 growth arrest in human fibroblasts. Cell Biol Int.

[11] Re FC, Manenti G, Borrello MG, Colombo MP, Fisher JH, Pierotti MA (1992). Multiple molecular alterations in mouse lung tumors. Mol Carcinog.

[12] Evtimova V, Zeillinger R, Weidle UH (2003). Identification of genes associated with the invasive status of human mammary carcinoma cell lines by transcriptional profiling. Tumour Biol.

[13] Mimori K, Kataoka A, Yoshinaga K, Ohta M, Sagara Y, Yoshikawa Y (2005). Identification of molecular markers for metastasis-related genes in primary breast cancer cells. Clin Exp Metastasis.

[14] Remondini D, O'Connell B, Intrator N, Sedivy JM, Neretti N, Castellani GC (2005). Targeting c-Myc-activated genes with a correlation method: detection of global changes in large gene expression network dynamics. Proc Natl Acad Sci U S A.

[15] van Dartel M, Hulsebos TJ (2004). Characterization of PMP22 expression in osteosarcoma. Cancer Genet Cytogenet.

[16] Huhne K, Park O, Liehr T, Rautenstrauss B (1999). Expression analysis of the PMP22 gene in glioma and osteogenic sarcoma cell lines. J Neurosci Res.

[17] Liu S, Chen Z (2015). The Functional Role of PMP22 Gene in the Proliferation and Invasion of Osteosarcoma. Med Sci Monit.

[18] Tawk M, Makoukji J, Belle M, Fonte C, Trousson A, Hawkins T (2011). Wnt/beta-catenin signaling is an essential and direct driver of myelin gene expression and myelinogenesis. J Neurosci.

[19] Both J, Wu T, Bras J, Schaap GR, Baas F, Hulsebos TJ (2012). Identification of novel candidate oncogenes in chromosome region 17p11.2-p12 in human osteosarcoma. PLoS One.

[20] van Dartel M, Cornelissen PW, Redeker S, Tarkkanen M, Knuutila S, Hogendoorn PC (2002). Amplification of 17p11.2 approximately p12, including PMP22, TOP3A, and MAPK7, in high-grade osteosarcoma. Cancer Genet Cytogenet.

[21] van Dartel M, Leenstra S, Troost D, Hulsebos TJ (2003). Infrequent but high-level amplification of 17p11.2 approximately p12 in human glioma. Cancer Genet Cytogenet.

[22] Cai W, Chen G, Luo Q, Liu J, Guo X, Zhang T (2017). PMP22 Regulates Self-Renewal and Chemoresistance of Gastric Cancer Cells. Mol Cancer Ther.

[23] Bartel DP (2004). MicroRNAs: genomics, biogenesis, mechanism, and function. Cell.

[24] Bartel DP (2009). MicroRNAs: target recognition and regulatory functions. Cell.

[25] Nelson KM, Weiss GJ (2008). MicroRNAs and cancer: past, present, and potential future. Mol Cancer Ther.

[26] Lau P, Verrier JD, Nielsen JA, Johnson KR, Notterpek L, Hudson LD (2008). Identification of dynamically regulated microRNA and mRNA networks in developing oligodendrocytes. J Neurosci.

[27] Verrier JD, Lau P, Hudson L, Murashov AK, Renne R, Notterpek L (2009). Peripheral myelin protein 22 is regulated post-transcriptionally by miRNA-29a. Glia.

[28] He X, Yu Y, Awatramani R, Lu QR (2012). Unwrapping myelination by microRNAs. Neuroscientist.

[29] Raymond CK, Roberts BS, Garrett-Engele P, Lim LP, Johnson JM (2005). Simple, quantitative primer-extension PCR assay for direct monitoring of microRNAs and short-interfering RNAs. Rna.

[30] Li Z, Hou J, Sun L, Wen T, Wang L, Zhao X (2012). NMI mediates transcription-independent ARF regulation in response to cellular stresses. Mol Biol Cell.

[31] Dai S, Wang X, Li X, Cao Y (2015). MicroRNA-139-5p acts as a tumor suppressor by targeting ELTD1 and regulating cell cycle in glioblastoma multiforme. Biochem Biophys Res Commun.

[32] Shao Q, Zhang P, Ma Y, Lu Z, Meng J, Li H (2018). MicroRNA-139-5p affects cisplatin sensitivity in human nasopharyngeal carcinoma cells by regulating the epithelial-to-mesenchymal transition. Gene.

[33] Wang Y, Li J, Xu C, Zhang X (2018). MicroRNA-139-5p Inhibits Cell Proliferation and Invasion by Targeting RHO-Associated Coiled-Coil-Containing Protein Kinase 2 in Ovarian Cancer. Oncol Res.

[34] Choi J, Kim YK, Park K, Nah J, Yoon SS, Kim DW (2016). MicroRNA-139-5p regulates proliferation of hematopoietic progenitors and is repressed during BCR-ABL-mediated leukemogenesis. Blood.

[35] Hou J, Wang T, Xie Q, Deng W, Yang JY, Zhang SQ (2016). N-Myc-interacting protein (NMI) negatively regulates epithelial-mesenchymal transition by inhibiting the acetylation of NF-kappaB/p65. Cancer Lett.

[36] Hou J, Jiang S, Zhao J, Zhu D, Zhao X, Cai JC (2017). N-Myc-Interacting Protein Negatively Regulates TNF-alpha-Induced NF-kappaB Transcriptional Activity by Sequestering NF-kappaB/p65 in the Cytoplasm. Sci Rep.

[37] Guo J, Miao Y, Xiao B, Huan R, Jiang Z, Meng D (2009). Differential expression of microRNA species in human gastric cancer versus non-tumorous tissues. J Gastroenterol Hepatol.

[38] Krishnan K, Steptoe AL, Martin HC, Pattabiraman DR, Nones K, Waddell N (2013). miR-139-5p is a regulator of metastatic pathways in breast cancer. RNA.

[39] Li RY, Chen LC, Zhang HY, Du WZ, Feng Y, Wang HB (2013). MiR-139 inhibits Mcl-1 expression and potentiates TMZ-induced apoptosis in glioma. CNS Neurosci Ther.

[40] Emmrich S, Engeland F, El-Khatib M, Henke K, Obulkasim A, Schoning J (2016). miR-139-5p controls translation in myeloid leukemia through EIF4G2. Oncogene.

[41] Zhu W, Zhu N, Bai D, Miao J, Zou S (2014). The crosstalk between Dectin1 and TLR4 via NF-kappaB subunits p65/RelB in mammary epithelial cells. Int Immunopharmacol.

[42] Zhang SQ, Kovalenko A, Cantarella G, Wallach D (2000). Recruitment of the IKK signalosome to the p55 TNF receptor: RIP and A20 bind to NEMO (IKKgamma) upon receptor stimulation. Immunity.

[43] Liu GH, Qu J, Shen X (2008). NF-kappaB/p65 antagonizes Nrf2-ARE pathway by depriving CBP from Nrf2 and facilitating recruitment of HDAC3 to MafK. Biochim Biophys Acta.

